# Fixation Methods in Primary Hip Arthroplasty: A Nationwide, Registry-Based Observational Study in Romania (2001–2024)

**DOI:** 10.3390/healthcare13192452

**Published:** 2025-09-27

**Authors:** Flaviu Moldovan, Liviu Moldovan

**Affiliations:** 1 Orthopedics—Traumatology Department, Faculty of Medicine, George Emil Palade University of Medicine, Pharmacy, Science, and Technology of Targu Mures, 540142 Targu Mures, Romania; 2 Faculty of Engineering and Information Technology, George Emil Palade University of Medicine, Pharmacy, Science, and Technology of Targu Mures, 540142 Targu Mures, Romania; liviu.moldovan@umfst.ro

**Keywords:** hip joint surgery, cemented, uncemented, hybrid, orthopedics surgery, osteoporosis, public health

## Abstract

Background/Objectives: Arthroplasty registries provide a broad database that constitutes evidence for discussions about cemented versus uncemented fixations. The objective of this study is to determine the current trend in fixation of total hip arthroplasties. Methods: From the Romanian Arthroplasty Register we extracted data regarding primary hip replacement surgery and revisions. We established evaluation variables and methodologies that contain volumes, variation trends, and gradients for surgical procedures, fixations and revision burdens. Results: In the period 2001–2024, the share of uncemented fixations was 56.8%, and that of cemented ones was 43.13%. The uncemented fixation gradient showed an increase from 0.32 in 2001 to 3.43 in 2024. We found an annual increase in the share of uncemented fixations (2.08%), to the detriment of cemented fixations, which decreased (−6.97%). We found that there is an obvious trend towards uncemented fixation, which is also evident in the elderly age group of 80+ years. The results regarding revision burdens remain within a relatively narrow range of 5.09–7.23%. The revision burdens of uncemented fixations are lower, ranging between 4.82% and 5.36%, compared to cemented fixations. Also, the revision burdens of cemented fixations have a decreasing trend of 0.54%. Conclusions: The variation trend of total uncemented implants is almost double compared to the variation trend of all primary hip joint surgeries. This indicates a trend towards uncemented fixation, and its share is increasing in all age groups. The increase in the proportion of uncemented fixations was associated with a small, non-significant decrease in revision burden.

## 1. Introduction

The most common indication for total hip arthroplasty (THA) is osteoarthritis [1]. The majority of patients suffering from osteoarthritis are over 60 years of age [2]. The prevalence of the diagnosis is higher in women than in men [3]. Another common cause of total hip arthroplasty is femoral neck fractures [4], found mainly in the cohort of patients over 80 years old [5]. In Romania, from 2008 to 2018, the number of hip fractures increased significantly by 53% in women and 22.4% in men [6]. Over 90% of fractures are fragility fractures. The crude incidence of low-energy hip fractures was 225/100,000 in women and 103/100,000 in men. This incidence is increases with age, reaching a peak rate of 1902/100,000 in women over 85 years of age [7].

THA significantly reduces pain and improves hip function and quality of life [8]. It is also the most commonly used procedure for treating avascular necrosis of the femoral head [9]. The volume of primary and revision interventions of the hip is estimated to double by 2034. Therefore, THA has become an increasingly common procedure [10], which also implies the risk of an increasing number of revision procedures [11].

The number of deaths in the perioperative period is significantly higher in patients undergoing cemented THA [12]. There is no difference in the risk of re-revision between cemented and uncemented stems after revisions performed for a periprosthetic femoral fracture treated with THA [13]. In general, uncemented intervention have a lower revision rate and risk, requiring “minor” procedures compared to hybrid ones [14].

Determining the optimal timing for the revision decision is affected by a number of factors that affect the accuracy of the process [15]. Of these, computer-assisted interventions are known to confer no substantial advantage in terms of attrition or survival rates [16,17]. The use of incompatible components from multiple manufacturers in THA has no adverse effects on outcomes [18]. The use of cemented fixations with a thick liner in the correct center of rotation appears to be the appropriate strategy for preventing wear of the polyethylene liner [19]. Uncemented collared stems are beneficial in decreasing the rate of intraoperative fractures without increasing the rate of subsidence or loosening [20]. Collared and long femoral stems have lower complication rates compared to collarless and short ones [21]. When using the uncemented Corail stem, surgeons can expect good results with a follow-up period of up to 30 years [22]. Patients with displaced femoral neck fractures treated with arthroplasty using a cemented stem show poor short-term functional outcomes [23]. For patients treated with uncemented hemiarthroplasty, the reduction in bone mineral density is more likely to be less intense in the contralateral hip and distal femur [24]. Thus, cemented fixation is the current technique for most patients with displaced femoral neck fractures treated with hemiarthroplasty [25]. The younger age group has a lower revision rate for uncemented versions than for hybrid versions [26]. In patients under 65 years of age, uncemented hip revisions have the lowest rate of aseptic loosening [27].

Recently, there has been a trend towards uncemented fixation in hip joint surgery [28]. Similar shifts have been reported internationally, including in England, Wales, Northern Ireland, and the Isle of Man [29]. Cemented fixation is considered an outdated practice in certain interventions. In these circumstances, the problem arises of identifying the fixation method that ensures the longest implant survival and is associated with the lowest risk of revision. This issue can be analyzed using statistical data from the national arthroplasty registry. Registry-based studies provide robust evidence for fixation outcomes [30]. For these reasons, we formulated the following hypotheses:

**H1:**  *In hip joint primary surgeries, there is an annual trend of increasing the share of uncemented fixations and a decreasing share of cemented fixations*.

**H2:**  *The proportion of uncemented fixations is increasing in all patient age groups.*

**H3:**  *As the proportion of uncemented fixations increases, the revision burden decreases.*

The aim of this study was to analyze national hip arthroplasty data to clarify fixation trends and their clinical implications in Romania. Specifically, we pursued three objectives:

1. To evaluate temporal trends in the use of cemented, uncemented, and hybrid fixation in primary hip arthroplasty (2001–2024).
2. To determine whether the increase in uncemented fixation is consistent across patient age groups.

These objectives were investigated using the Romanian Arthroplasty Register, which provides complete national coverage and enables longitudinal analysis of fixation practices.

## 2. Materials and Methods

### 2.1. Study Design and Participants

This was a retrospective, registry-based observational study. Data were obtained from the Romanian Arthroplasty Register (RAR) [31], which has collected nationwide arthroplasty data since 2001. Reporting to RAR is mandatory for all 125 orthopedic clinics/departments in Romania according to the Ordinance of the Minister of Health No. 1591/1110/2010, ensuring full national coverage.

Inclusion criteria: All consecutive patients undergoing primary or revision hip arthroplasty between 1 January 2001 and 31 December 2024, irrespective of age or sex, were included.

Exclusion criteria: Procedures performed outside this period or not reported to the RAR were excluded. Because reporting is mandatory, the extent of missing data is minimal. No additional exclusion criteria (e.g., by diagnosis or implant type) were applied, reflecting the population-based nature of the registry.

We used all data reported in RAR for the following interventions:

- Hip replacement surgery, detailed by subsequent surgeries: total hip arthroplasty— code O12104 (further detailed into prostheses: cemented total hip arthroplasty, uncemented total hip arthroplasty, hybrid, and reverse hybrid total hip arthroplasty), bipolar hemiarthroplasty, unipolar hemiarthroplasty—Moore type—code O12103. We also extracted the gender of the patients who underwent these interventions.
- Revision hip arthroplasty—code O12401.

A total of 222,462 subjects were included in the study, which were registered in RAR (Table 1). The raw annual counts in the period 2001–2024, for primary surgery, revision surgery, cemented, uncemented, and hybrid fixations are provided in the Supplementary Materials.

Patient consent was obtained for data collection and inclusion in RAR. According to the specifications of the Ministry of Health, separate informed consent and ethical approval were not required for the present study.

This study followed the STROBE (Strengthening the Reporting of Observational Studies in Epidemiology) guidelines for reporting observational studies, as recommended by the EQUATOR network [32]. The completed STROBE checklist is provided in the Supplementary Materials.

### 2.2. Evaluation Variables and Methodologies

Based on the formulated hypotheses, we have defined the variables and developed evaluation methodologies appropriate to the following explored issues: the volumes of surgical procedures, fixation type, and revision burden.

#### 2.2.1. The Volumes of Surgical Procedures and Variation Trends

To estimate the tendency for the surgery procedures volumes, we selected as variables the annual numbers of the following interventions: hip primary joint surgery (nHPjs), hip revision joint surgery (nHRjs), hip primary joint surgery total cemented (nHPjsTC), hip primary joint surgery total uncemented (nHPjsTU), and hip primary joint surgery total hybrid (nHPjsTH).

We estimated the trends by calculating the arithmetic mean of the percentage differences in the volumes of interventions between two successive years (y + 1) and (y), relative to the base year (y). We performed the calculations for all categories of interventions studied.

In the case of hip primary joint surgery, the variation trend is expressed in the form:

(1) $${VT}_{HPjs}=\sum_{y=1}^{Y}\frac{nHPjs\left(y+1\right)-nHPjs(y)}{Y\times nHPjs(y)}\cdot 100\;[\%]$$

where nHPjs(y) is the number of hip replacement surgeries related to year (y) in the interval 2001–2024. We repeated the calculation without considering the pandemic years by excluding values from the 2020–2023 range. Similar formulas were used for the other categories of interventions: hip joint revision surgery, hip joint primary surgery total cemented, and hip joint primary surgery total uncemented.

To estimate the trend of using uncemented versus cemented fixations, we created a scenario in which we distributed 0.5 of the hybrid fixations to each of the two fixation types and calculated their equivalent number:

(2) $${\left(nHPjsTC\right)}^{equ}=nHPjsTC+\frac{1}{2}\times \left(nHPjsTH\right)$$

and:

(3) $${\left(nHPjsTU\right)}^{equ}=nHPjsTU+\frac{1}{2}\times \left(nHPjsTH\right)$$

For hybrid prostheses, we distributed half of the cases to cemented and half to uncemented fixation categories. This 0.5/0.5 allocation reflects the fact that hybrids contain one cemented and one uncemented component and avoids introducing bias toward either fixation method. For hemiarthroplasty, we applied weights derived from national registry reports: bipolar prostheses were split approximately equally between cemented (0.501) and uncemented (0.499), while unipolar prostheses were overwhelmingly uncemented (0.955 uncemented; 0.045 cemented), consistent with Romanian practice patterns in elderly patients. To test robustness, we repeated analyses under alternative allocation scenarios (cemented-biased and uncemented-biased).

We defined the uncemented fixation gradient (UFG) as the ratio of the equivalent number of uncemented fixations to the equivalent number of cemented fixations:

(4) $$UFG=\frac{{\left(nHPjsTU\right)}^{\mathrm{equ}}}{{\left(nHPjsTC\right)}^{\mathrm{equ}}}$$

#### 2.2.2. Revision Burden

For the study of revision burdens, we selected the percentage of arthroplasty revisions as the variable. It was computed as the ratio between the number of revision arthroplasties and the number of primary arthroplasties in the same period multiplied by 100. In this way, we calculated the hip revision burden:

(5) $$RB=\frac{nHRjs}{nHPjs}\cdot 100[\%]$$

By describing the proportions of hip arthroplasties that required revision surgery, surgeons have insight into the reliability of the interventions performed.

#### 2.2.3. Correlation Between Fixation Type and Revision Burden

To study the influence of fixation mode on revision burden in the period 2001–2024, we represented the correlation between the uncemented fixation gradient and revision burden. The two variables are defined by relations (4) and (5). A positive correlation indicates an increase in revision tasks with an increase in the number of uncemented fixations, and a negative correlation indicates a decrease.

### 2.3. Data Collection and Statistical Analysis

Data were collected in June 2025, from the “National Hip Joint Surgery Statistics” section of the Romanian Arthroplasty Register website. From the presented linear and column graphs, which indicate the number of cases per year, we extracted the volumes of the studied intervention categories into Excel files. For this, we examined the annual trend in the number of surgeries corresponding to the period 2001–2024.

The data were filtered, analyzed primarily, and transferred to Microsoft Excel, GNU PSPP, and Matlab for further processing. Statistical analysis was performed using SPSS–IBM (SPSS, Inc., Chicago, IL, USA) for Windows version 29.0.2 and Excel (Microsoft 365, Albuquerque, NM, USA) version 2508 (Build 19127.20264). We employed a multi-faceted statistical approach that consisted of a simple linear regression analysis using annual fixation shares as the dependent variable and calendar year as the independent variable. To investigate the relationship between patient age groups and the use of uncemented fixation, we applied Spearman’s rank correlation coefficient (ρ). This non-parametric test was chosen due to the ordinal nature of age groups and the monotonic pattern expected. To explore the relationship between the gradient of uncemented fixation usage and revision burden, we utilized Pearson’s correlation coefficient (r). This test was appropriate given the continuous nature of both variables. Significance was set at *p* < 0.05.

To assess the validity of the linear regression models, we conducted diagnostic checks for autocorrelation in the residuals. The Durbin–Watson test was applied to all regression models. In addition, residual plots were visually inspected to detect systematic deviations. As a robustness check, we also repeated the analyses using Prais–Winsten regression, which corrects for potential first-order autocorrelation, to ensure that serial dependence did not bias the estimated trends.

## 3. Results

In our study, we addressed the data of the 125 orthopedic clinics/departments in Romania which must report their arthroplasty activity (hip, knee, and spine) to the Romanian Arthroplasty Register, according to the Ordinance of the Minister of Health No 1591/1110/2010. It represents 100% of the orthopedic clinics/departments existing at the national level.

### 3.1. Hip Primary Joint Replacement Surgeries by Fixation

We extracted the available data from the RAR regarding the fixation type of hip joint replacement surgery: total cemented, total uncemented, or total hybrid (Table 2).

**Figure 1:**
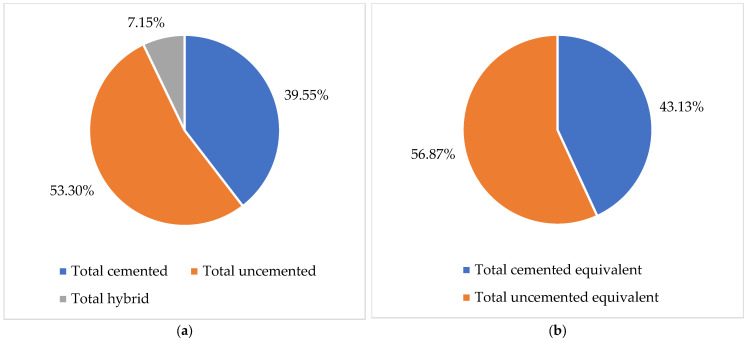
The percentage shares of hip replacement surgeries by fixation in the period 2001–2024: (**a**) actual values; (**b**) equivalent values.

With the support of the data from Table 2, we represented in Figure 1 the percentages of hip primary joint surgeries by fixation for patients in the period 2001–2024. The analysis shows that the largest share is total uncemented fixation 53.30%, followed by total cemented 39.55%, and total hybrid 7.15%.

**Figure 2:**
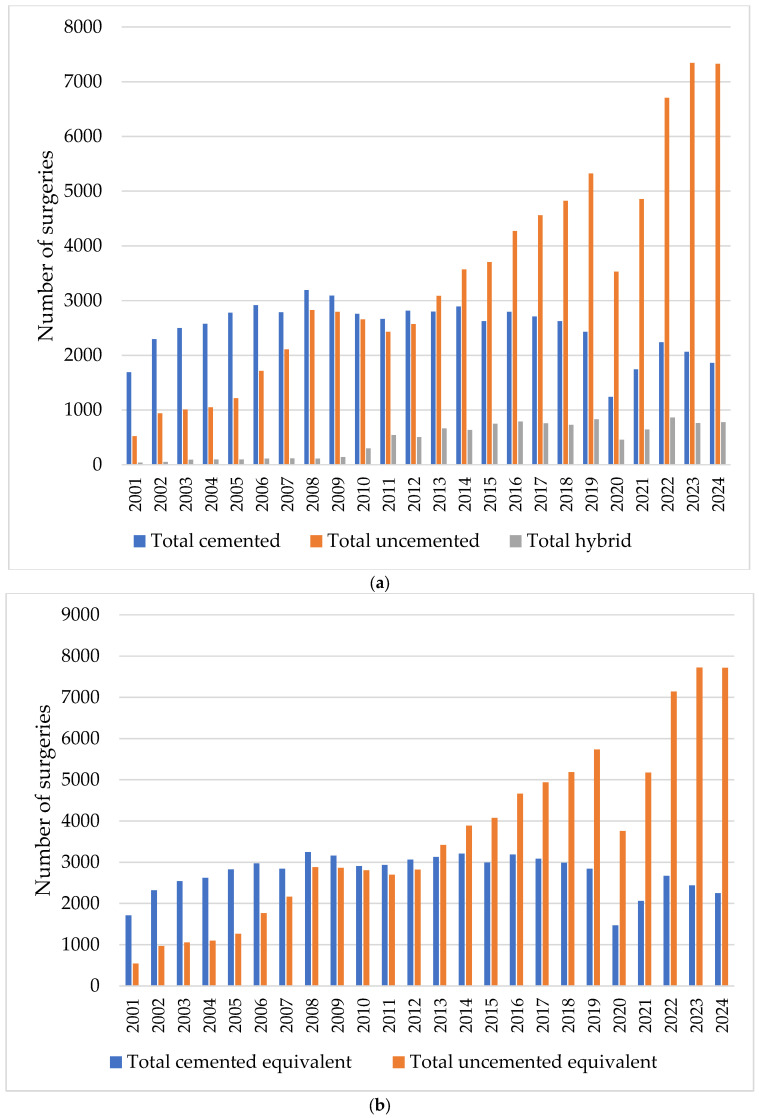
Prevalence of annual hip primary joint replacement surgeries between 2001 and 2024 by fixation: (**a**) actual interventions; (**b**) equivalent interventions.

In the continuation of the study, we examined the annual trend in the volume of cases between 1 January 2001 and 31 December 2024 for actual hip primary joint surgeries by fixation: total cemented, total uncemented, and total hybrid; and for equivalent hip primary joint surgeries by fixation: total cemented and total uncemented (Figure 2).

In both situations, the cemented interventions showed a slight upward trend until 2008, after which the decrease is evident. In contrast, the trend of increasing uncemented interventions is much more pronounced.

**Figure 3:**
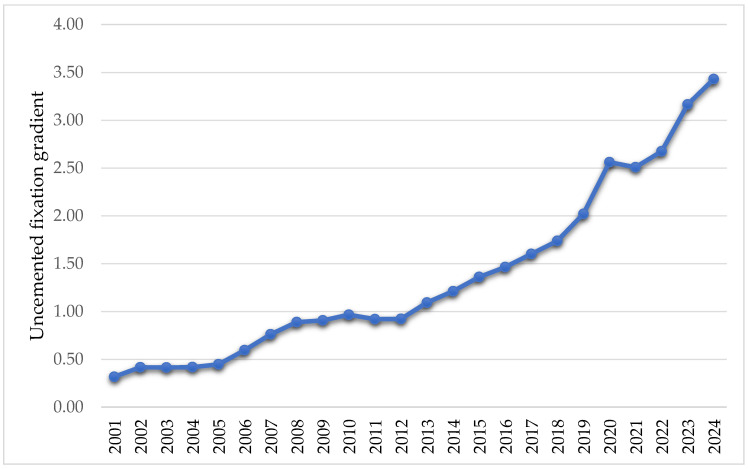
Variation of uncemented fixation gradient in the period 2001–2024.

The evolution of the uncemented fixation gradient, defined as the ratio between the total number of equivalent uncemented fixations and the total number of equivalent cemented fixations from the period 2001–2024, shows a continuous increase, from 0.32 in 2001 to 3.43 in 2024 (Figure 3).

We calculated the variation trends of the hip primary surgeries studied, with Formula (1) for Y = 24 years, corresponding to the interval (2001–2024), which also includes the pandemic period (Table 3). We performed the same calculations without the pandemic period, for Y = 20 years (2001–2019 and 2024).

As shown in Table 3, the annual growth rates for revision surgeries (HRjs) is 8.58%, which is higher than that for primary joint surgeries (HPjs) at 7.35%. Among actual primary joint surgery by fixation, the highest annual growth rates, in descending order, are for total hybrid (HPjsTH) 19.80%, total uncemented (HPjsTU) 2.08%, and total cemented (HPjsTC) −3.83%. In the case of equivalent primary joint surgery by fixation, the highest growth rate is for total uncemented ${\left(HPjsTC\right)}^{equ}$ at 14.14%, which is 2.84 times higher than that for total cemented.

By calculating the annual growth rate, we demonstrated that some fixation interventions, such as total uncemented and total hybrid hip primary joint surgeries, are increasing, and others such as total cemented, are decreasing. Total cemented fixations register negative actual values and positive equivalent values. Total uncemented fixations register positive actual and equivalent values. The finding of an annual increase in the share of uncemented fixations and a decrease in the share of cemented fixations in hip primary joint surgeries indicates a trend towards uncemented fixations. With this support, the volumes and types of fixations that will be carried out in the coming period in Romania can be predicted.

### 3.2. Implant Distribution by Age Groups

**Figure 4:**
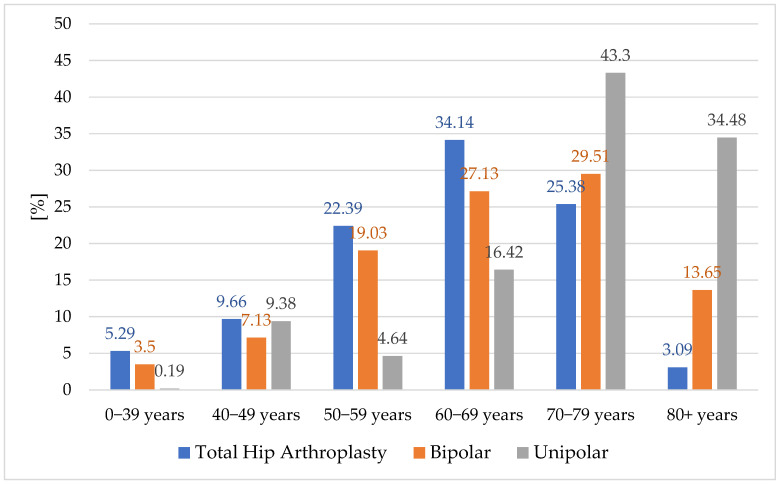
Hip primary implant distribution by age groups.

The most recent statistics from the Romanian Arthroplasty Registry [33] regarding hip implants by age group indicate that, for young and middle-aged people, the most common type of implant is the total hip prosthesis. This trend has the tendency to decrease with the older age groups (Figure 4).

**Figure 5:**
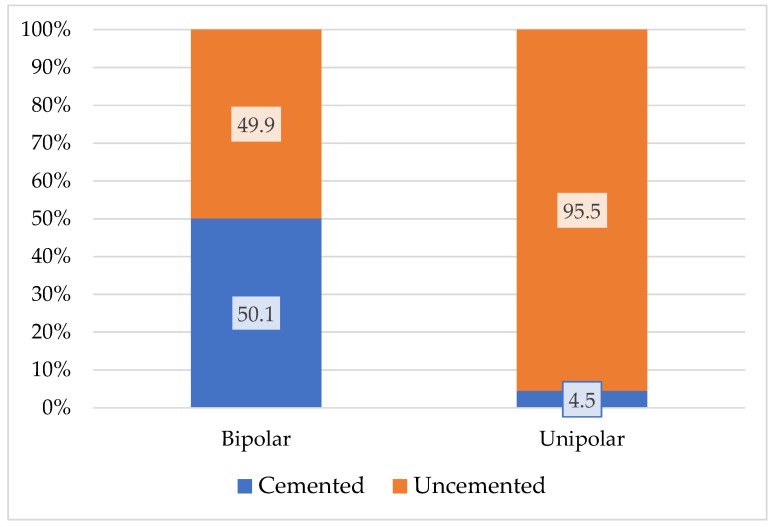
Distribution of fixation type by primary implant type.

The highest proportion is 34.14% for the age group 60–69 years. As expected, the highest figures for unipolar-type implants, including Moore and Thompson, are in the age group including patients over 70 years old, with a ratio of 43.3% for the age group 70–79 years. The presence of unipolar implants in age groups under 70 years old is explained only by social and economic criteria and cannot be explained for medical reasons. Bipolar implants, reach a maximum in age groups 60–79 years. The distribution of fixation type per primary implant type for bipolar and unipolar type according to the same RAR statistics [34] is shown in Figure 5.

**Figure 6:**
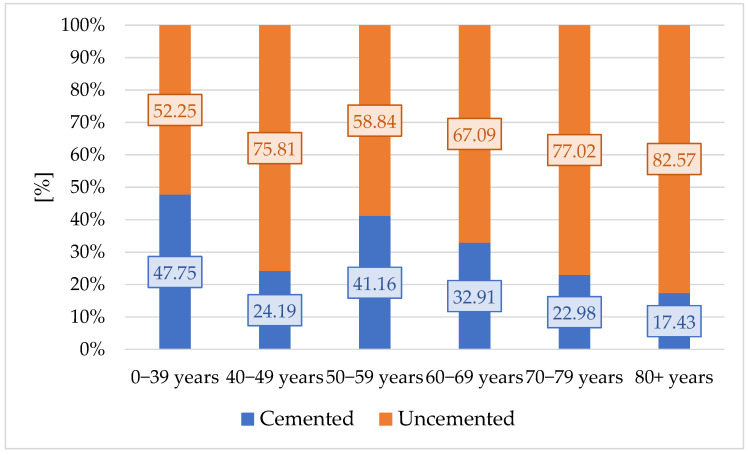
Equivalent fixations of bipolar and unipolar prostheses by age groups.

We then used these results and equated a bipolar prosthesis with 0.501 cemented prostheses and 0.499 uncemented prostheses. We equated the unipolar prosthesis with 0.045 cemented prostheses and 0.955 uncemented prostheses. Equivalent fixations of bipolar prostheses and unipolar prostheses per age groups indicate an increase in the proportion of uncemented interventions with age (Figure 6).

The proportion of uncemented fixations increases continuously with age, from 52.25% in the 0–39 years group to 82.57% in the 80+ years group. The trend of annual decrease in the share of total cemented fixations and increase in the share of total uncemented fixations of bipolar and unipolar type implants is also confirmed in older age groups.

### 3.3. Revision Burden

**Figure 7:**
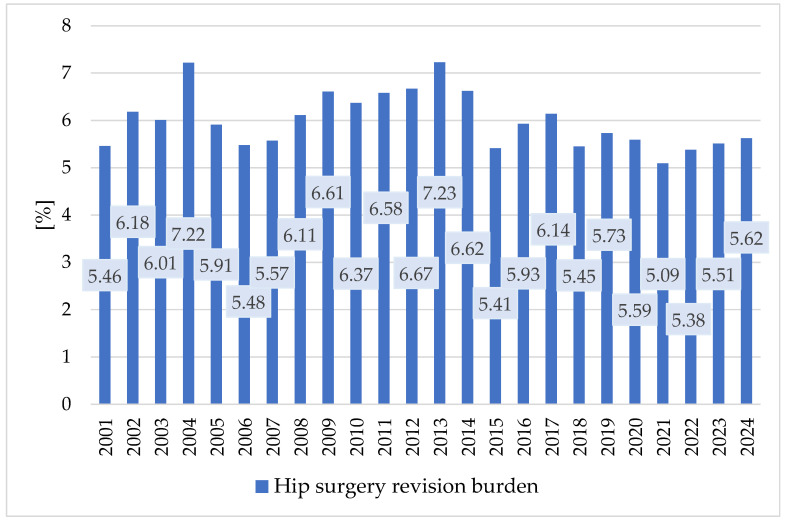
Variation of hip surgery revision burden at the national level in the period 2001–2024.

The results regarding the hip arthroplasty revision burdens at the national level are presented in Table 4, and their variations are represented in Figure 7.

Revision burdens were 5.09–7.23% for the hip joint. The increasing trend in the number of revision interventions was manifested mainly in the period 2001–2013. After that, they decreased, especially during the pandemic.

**Figure 8:**
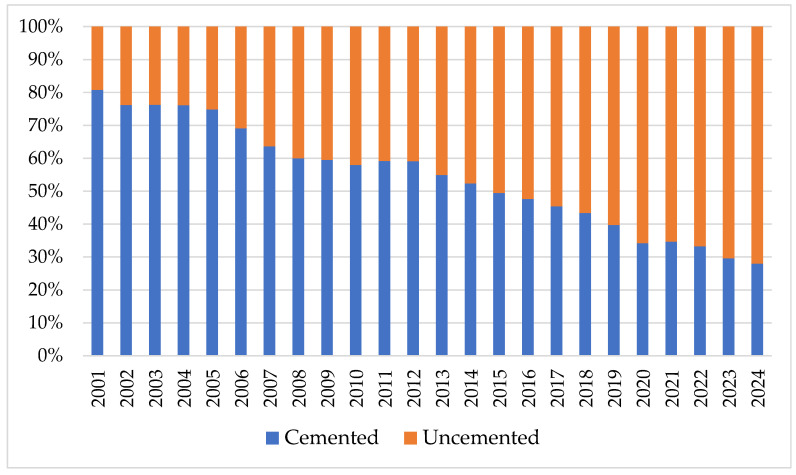
Revision burden shares by the type of fixation.

Figure 8 shows the variation in the shares of cemented and uncemented revisions. In 2001, uncemented interventions had a share of 24.05% in total interventions and recorded 19.23% of the total revision burden. In 2024, the share of uncemented interventions represented 77.42% with a share in revision burden at 72.06%. The intermediate values in the analyzed period fall between these limits.

**Figure 9:**
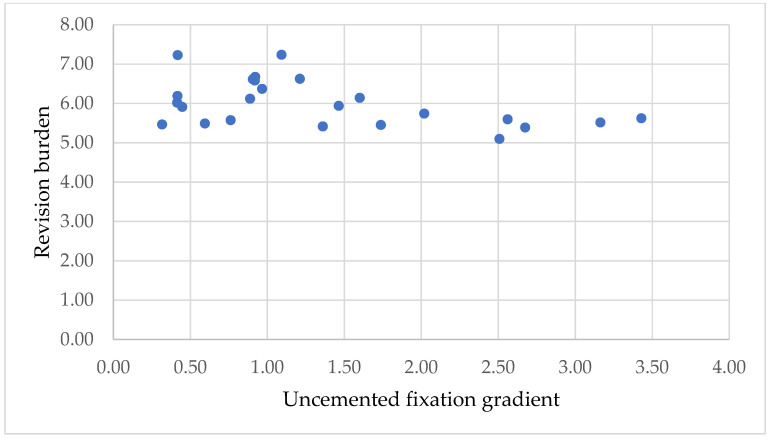
Correlation between uncemented fixation gradient and revision burden.

We represented the correlation between uncemented fixation gradient and revision burden in the interval 2001–2024, which may provide information regarding the influence of fixation mode on revision burden (Figure 9).

There is a slight negative correlation between the two variables. This result indicates that with the increase in the number of uncemented fixations, there is a slight decrease in the revision burden.

We conducted formal statistical tests to evaluate the hypotheses presented in this study:

H1: Linear regression of fixation shares over time confirmed a significant upward trend in uncemented fixations (slope = +2.09%/year, *p* < 0.0001) and a significant downward trend in cemented fixations (slope = −2.48%/year, *p* < 0.0001).

Regression diagnostics confirmed that the assumptions of independence were not violated. The Durbin–Watson statistic was 1.95 for the uncemented fixation model and 2.08 for the cemented fixation model, both values close to 2, indicating no significant autocorrelation. Visual inspection of residuals revealed no systematic patterns. Sensitivity analysis with Prais–Winsten regression yielded nearly identical results to the OLS models (uncemented fixation slope = +2.01%/year, *p* < 0.0001; cemented fixation slope = −2.44%/year, *p* < 0.0001). These findings support the robustness of the reported trends.

H2: Spearman correlation analysis showed a perfect monotonic increase in uncemented fixations across age groups (ρ = 1.0, *p* < 0.0001), confirming that the proportion of uncemented fixations increases with patient age.

H3: Pearson correlation between the uncemented fixation gradient and revision burden yielded a moderate negative correlation (r = −0.37) but was not statistically significant (*p* = 0.079). This suggests a potential inverse relationship, though further evidence is needed.

These statistical results support the conclusions of our study regarding the fixation trends in hip arthroplasty in Romania. To assess the robustness of the allocation method, we repeated the analysis using alternative distributions of hybrid and hemiarthroplasty prostheses. In Scenario A, 70% of these cases were assigned to cemented fixation; in Scenario B, 70% were assigned to uncemented fixation. Across both scenarios, the direction and significance of the fixation trends remained unchanged: uncemented fixations continued to increase significantly over time, and cemented fixations decreased significantly (all *p* < 0.0001). Thus, the main findings are not dependent on the exact allocation coefficients.

## 4. Discussion

The analysis of hip joint surgery statistics between 2001 and 2024 indicates that in terms of fixation, the interventions are 53.30% total cemented, 39.55% total uncemented, and 7.13% total hybrid. By equating hybrid interventions and classifying them into the other two categories, we showed that 56.8% of fixations are uncemented, and 43.13% are cemented. Like the findings of the study conducted by Hameed et al. [35], we found a trend towards uncemented interventions. This is consistent with large registry reports from the UK [29], Nordic countries [30], and the US [36]. These increased from 541.5 interventions in 2001 to 7716 interventions in 2014. Comparatively, during the same period, cemented interventions decreased from 2250to 1710.5. Also, the uncemented fixation gradient, the specific indicator we created in this research, showed an increase from 0.32 in 2001 to 3.43 in 2024.

The variation trends of hip primary and hip revision surgeries in Romania indicate positive values for hip primary joint surgery of 7.51%, and hip revision joint surgery of 8.58%. This means a greater increase in the revision rate compared to the primary intervention rate, a finding similar to that of the study carried out by Pamilo et al. [37]. Uncemented fixations increased steadily, while cemented fixations declined, confirming a clear shift toward uncemented arthroplasty. The result reveals the trend in hip joint surgery towards uncemented fixation, which is also supported by the study conducted by Moore et al. [38]. The finding allows the prediction of the volumes and types of fixations that will be needed soon in hip primary joint surgeries, as well as the provision of adequate material and human resources.

The analysis of interventions by age indicates a maximum of THA for the 60–69 years group and many bipolar and unipolar (Moore and Thompson type) interventions for the older age groups over 70 years. Equivalent prosthesis fixations according to the methodology in this study indicate that there is a clear trend towards uncemented fixation, although this is not recommended by some studies [39]. This also manifests itself in older age groups. In the 0–39 years group, 47.75% of fixations are cemented and 52.25% of fixations are uncemented. The difference between the two fixation methods continues to increase, so that in the 80+ years age group, it records a maximum of 17.43% for cemented fixations and 82.57% for uncemented fixations. In the analysis by age group, this result confirms the H1 hypothesis that in primary hip joint surgeries, there is an annual trend of increasing shares of uncemented fixations and a decrease in shares of cemented fixations.

The results regarding the hip arthroplasty revision burdens at national level remain within a relatively narrow range of 5.09–7.23%, with a slight downward trend after 2014. The higher proportion of uncemented fixations may explain this variation through the longer duration at which these types of fixations require revision. Compared to cemented fixations, the revision burdens of uncemented fixations are lower, ranging between 4.82% and 5.36%. During the study period, a slight decrease of approximately 0.54% in revision burdens of cemented fixations was observed. The result agrees with studies conducted in Korea [40], the United States [41], Germany [42], and Finland [43]. It also aligns with a systematic review of registry data that confirmed favorable long-term outcomes for uncemented stems [44]. Together, these results confirm a nationwide shift toward uncemented fixation across all age groups, with a modest association between greater uncemented use and lower revision burden.

### 4.1. Perspectives for Clinical Practice

The findings of this study have direct implications for clinical practice in Romania and internationally [45]. The observed trend toward uncemented fixation across all age groups, including elderly patients, suggests that orthopedic surgeons are increasingly prioritizing implants with favorable long-term survival and lower revision burdens. From a surgical perspective, this requires continuous training and dissemination of evidence-based techniques for uncemented arthroplasty, particularly in fragile elderly populations where cemented fixation has historically been preferred.

Beyond the operating room, hip arthroplasty care should be understood in a multidisciplinary and multidimensional context. Patients undergoing hip replacement often require coordinated management across the perioperative continuum, including anesthesiology, geriatrics, rehabilitation, nursing, and primary care. Effective clinical networks are crucial for integrating these services. Evidence shows that clinical networks can support the achievement of the quadruple aim by simultaneously improving patient outcomes, population health, professional satisfaction, and cost efficiency [46].

Similarly, the implementation of standardized care pathways has been shown to reduce variability, enhance patient safety, and improve efficiency in surgical care [47,48]. Embedding arthroplasty within such pathways—spanning preoperative optimization, intraoperative safety checklists, and postoperative rehabilitation—may help ensure that the advantages of uncemented fixation translate into tangible benefits for patients and health systems.

Finally, the integration of digital tools and new technologies—such as registry-based feedback systems, data-driven predictive analytics, and computer-assisted planning—can further strengthen decision-making, although their adoption should always be embedded within clinically validated pathways and coordinated networks [49].

Taken together, our results emphasize that the choice of fixation method should not be seen in isolation but as part of a holistic patient-care process, supported by structured clinical networks, standardized pathways, and technology-enabled solutions.

In addition to surgical decision-making, the management of hip arthroplasty patients should be understood within a broader framework of care delivery. The Chronic Care Model (CCM), widely applied across Europe, provides a multidimensional approach that emphasizes productive interactions between informed patients and proactive healthcare teams [50,51]. In the context of hip arthroplasty, CCM highlights the need for coordinated long-term management, including osteoporosis care, fall prevention, rehabilitation, and comorbidity management [52]. This perspective aligns with our findings, as the shift toward uncemented fixation should be accompanied by structured follow-up and continuity of care to maintain functional outcomes and minimize revision burden.

### 4.2. Limitations

However, our study has some limitations. A first limitation is the use of data from national registers in Romania, which, compared to other countries, have a limited amount of information. At the same time, some information is not collected by age groups. Specifically, the limitation is induced by the lack of accurate information regarding THA prior to 2001, when RAR began systematic data collection at the national level. The lack of pre-2001 data and the absence of functional outcomes and implant type details limit clinical interpretation. The correlation between the uncemented fixation gradient and revision burden does not reach statistical significance, which limits the clinical evidence without more robust evidence or controlled analyses. Another limitation comes from the way revision burden is calculated as the ratio between the number of revisions in arthroplasties and the number of primary arthroplasties in the same period. This does not consider follow-up time and may underestimate the true revision risk. The study is also limited because fixations are not recorded in the RAR by gender. However, sensitivity analyses indicate the robustness of the calculations performed. We have forecasted the annual growth rate for the studied interventions, which is applicable for the immediate future, but as more data are collected in the RAR, this can be updated for higher accuracy predictions.

Future research directions recommend diversifying data from the national registry, that will allow for new, more detailed research, numerical simulation, and automated data processing [53,54]. Another direction for research is to replicate the model in other countries to determine whether the fixation trend in hip fractures surgeries is similar.

## 5. Conclusions

In Romania between 2001 and 2024, in hip joint surgery 56.8% of fixations are uncemented and 43.13% are cemented. In the same period, the uncemented fixation gradient shows an increase from 0.32 to 3.43. This variation trend of totally uncemented hip arthroplasties is almost double compared to that of all hip primary joint surgeries, which indicates the trend. The proportion of uncemented fixations increases in all age groups of patients. The revision burdens of uncemented fixations are lower, ranging between 4.82% and 5.36%, compared to cemented fixations. The revision burdens of cemented fixations have a decreasing trend of 0.54%. While the negative correlation between uncemented fixation and revision burden did not reach statistical significance (*p* = 0.079), the trend is consistent with international findings, but the implication of clinical significance requires stronger evidence or controlled analyses.

The study findings allow the prediction of the volumes and types of fixations that will be needed soon in hip primary joint surgeries, as well as the provision of adequate material and human resources.

Beyond statistical trends, these findings should be interpreted within the broader framework of patient care. The choice of fixation methods must be embedded in multidisciplinary networks and standardized care pathways to ensure that the benefits of uncemented fixation translate into improved outcomes, patient safety, and system-wide efficiency. The management of hip arthroplasty patients should be understood within broader framework of the Chronic Care Model, which can serve as a model for Romania and other countries aiming to optimize hip arthroplasty care pathways. The shift toward uncemented fixation should be accompanied by structured follow-up and continuity of care to maintain functional outcomes and minimize revision burden.

## Figures and Tables

**Table 1:** Total number of patients in the period 2001–2024 included in the study.

Primary Hip Joint Surgery	Revision Hip Joint Surgery	Total Cemented Fixation	Total Uncemented Fixation	Total Hybrid Fixation
222,462	13,264	60,045	80,916	10,835

**Table 2:** The number and percentage shares of hip replacement surgeries by fixation in the period 2001–2024.

Year	2001	2002	2003	2004	2005	2006	2007	2008	2009	2010	2011	2012
Total cemented (n)	1690	2295	2495	2576	2778	2915	2784	3190	3089	2755	2663	2812
Total uncemented (n)	521	940	1006	1048	1216	1712	2106	2827	2792	2655	2426	2571
Total hybrid (n)	41	53	93	95	97	111	114	111	141	300	542	504
Total cemented [%]	75.04	69.80	69.42	69.27	67.91	61.52	55.64	52.06	51.30	48.25	47.29	47.77
Total uncemented [%]	23.13	28.59	27.99	28.18	29.72	36.13	42.09	46.13	46.36	46.50	43.08	43.67
Total hybrid [%]	1.82	1.61	2.59	2.55	2.37	2.34	2.28	1.81	2.34	5.25	9.63	8.56
Year	2013	2014	2015	2016	2017	2018	2019	2020	2021	2022	2023	2024
Total cemented (n)	2797	2892	2622	2791	2707	2623	2427	1240	1743	2238	2062	1861
Total uncemented (n)	3086	3571	3702	4269	4559	4824	5323	3529	4856	6707	7343	7327
Total hybrid (n)	661	633	748	789	754	725	828	454	643	862	758	778
Total cemented [%]	42.74	40.76	37.08	35.56	33.75	32.10	28.29	23.74	24.07	22.82	20.29	18.67
Total uncemented [%]	47.16	50.32	52.35	54.39	56.85	59.03	62.05	67.57	67.05	68.39	72.25	73.52
Total hybrid [%]	10.10	8.92	10.58	10.05	9.40	8.87	9.65	8.69	8.88	8.79	7.46	7.81

**Table 3:** The variation trends of hip primary and revision surgeries in Romania.

Surgical Intervention	Variation Trends	
	Including Pandemic [%]	Without Pandemic [%]
Hip primary joint surgery (HPjs)	7.51	7.35
Hip revision joint surgery (HRjs)	8.44	8.58
Hip primary joint surgery total cemented (HPjsTC)	−3.83	−6.97
Hip primary joint surgery total uncemented (HPjsTU)	2.08	1.78
Hip primary joint surgery total hybrid (HPjsTH)	18.02	19.80
Hip primary joint surgery total cemented − equivalent ${\left(HPjsTC\right)}^{\mathrm{equ}}$	4.94	2.56
Hip primary joint surgery total uncemented − equivalent ${\left(HPjsTU\right)}^{\mathrm{equ}}$	14.14	13.80

**Table 4:** Hip surgery revision burdens.

Year	2001	2002	2003	2004	2005	2006	2007	2008	2009	2010	2011	2012
Primary surgery (nHPjs)	3312	5236	5947	5922	6345	7141	7286	8974	8891	8869	8859	8973
Revision (nHRjs)	181	324	358	428	375	392	406	549	588	565	583	599
Revision burden (RB)	5.46	6.18	6.01	7.22	5.91	5.48	5.57	6.11	6.61	6.37	6.58	6.67
RB cemented	4.41	4.72	4.59	5.51	4.43	3.80	3.55	3.67	3.94	3.69	3.89	3.95
RB uncemented	1.05	1.47	1.43	1.72	1.48	1.69	2.02	2.45	2.67	2.68	2.69	2.73
Year	2013	2014	2015	2016	2017	2018	2019	2020	2021	2022	2023	2024
Primary surgery (nHPjs)	9814	10,401	10,546	11,554	11,705	11,944	12,179	8025	10,167	13,269	13,599	13,504
Revision (nHRjs)	710	689	571	686	719	651	699	449	518	715	750	759
Revision burden (RB)	7.23	6.62	5.41	5.93	6.14	5.45	5.73	5.59	5.09	5.38	5.51	5.62
RB cemented	3.97	3.47	2.68	2.83	2.79	2.37	2.28	1.92	1.77	1.79	1.64	1.57
RB uncemented	3.26	3.15	2.73	3.11	3.35	3.08	3.46	3.68	3.32	3.6	3.88	4.05

## Data Availability

Data are contained within the article.

## Supplementary Materials

The following supporting information can be downloaded at: https://www.mdpi.com/article/10.3390/healthcare13192452/s1, Table S1: The raw annual counts in the period 2001–2024, for primary surgery, revision surgery, cemented, uncemented, and hybrid fixations; Table S2: Completed STROBE checklist for this study.

## Abbreviations

The following abbreviations are used in this manuscript:

nHPjs	hip primary joint surgery
nHRjs	hip revision joint surgery
nHPjsTC	hip primary joint surgery total cemented
nHPjsTU	hip primary joint surgery total uncemented
nHPjsTH	hip primary joint surgery total hybrid
${\left(nHPjsTC\right)}^{equ}$	equivalent number of hip primary joint surgery total cemented
${\left(nHPjsTU\right)}^{equ}$	equivalent number of hip primary joint surgery total uncemented
UFG	uncemented fixation gradient
RB	hip revision burden
VT	variation trend
y	year
