# Supplementary material for: Fixation Methods in Primary Hip Arthroplasty: A Nationwide, Registry-Based Observational Study in Romania (2001–2024)
Source: Healthcare (Basel). 2025 Sep 27;13(19):2452. doi: 10.3390/healthcare13192452 (PMC12524635; doi:10.3390/healthcare13192452)
Supplement: Supplementary file 1 [file healthcare-13-02452-s001.zip › healthcare-3871992-supplementary.pdf]

SUPPLEMENTARY MATERIALS

Fixation Methods in Primary Hip Arthroplasty: A Nationwide, Registry-Based Observational Study in Romania (2001–2024)

Authors: Flaviu Moldovan[1,*] and Liviu Moldovan[2]

[1] Orthopedics—Traumatology Department, Faculty of Medicine, George Emil Palade University of Medicine, Pharmacy, Science, and Technology of Targu Mures, 540142 Targu Mures, Romania

2 Faculty of Engineering and Information Technology, George Emil Palade University of Medicine, Pharmacy, Science, and Technology of Targu Mures, 540142 Targu Mures, Romania; liviu.moldovan@umfst.ro

* Correspondence: flaviu.moldovan@umfst.ro; Tel.: +40 754 671 886

Table S1. The raw annual counts in the period 2001–2024, for primary surgery, revision surgery, cemented, uncemented, and hybrid fixations.

Year	**Primary hip joint surgery**	**Revision hip joint surgery**	**Total cemented fixation**	**Total uncemented fixation**	**Total hybrid fixation**
2001	3312	181	1690	521	41
2002	5236	324	2295	940	53
2003	5947	358	2495	1006	93
2004	5922	428	2576	1048	95
2005	6345	375	2778	1216	97
2006	7141	392	2915	1712	111
2007	7286	406	2784	2106	114
2008	8974	549	3190	2827	111
2009	8891	588	3089	2792	141
2010	8869	565	2755	2655	300
2011	8859	583	2663	2426	542
2012	8973	599	2812	2571	504
2013	9814	710	2797	3086	661
2014	10,404	689	2892	3571	633
2015	10,555	571	2622	3702	748
2016	11,565	686	2791	4269	789
2017	11,705	719	2707	4559	754
2018	11,944	651	2623	4824	725
2019	12,180	699	2427	5324	828
2020	8025	449	1240	3529	454
2021	10,167	522	1743	4856	643
2022	13,269	715	2238	6709	862
2023	13,602	751	2062	7348	758
2024	13,477	754	1861	7319	778
Total	222,462	13,264	60,045	80,916	10,853

Table S2. Completed STROBE checklist for this study.

Section/ Topic	Item No.	Recommendation	Reported on page(s)
Title and abstract	1	(a) Indicate the study's design with a commonly used term in the title or abstract. (b) Provide in the abstract an informative and balanced summary of what was done and what was found.	Title p.1; Abstract p.2
Introduction	2	Explain the scientific background and rationale for the investigation being reported.	pp.2–3
	3	State specific objectives, including any prespecified hypotheses.	p.4
Methods	4	Present key elements of study design early in the paper.	pp.4–5
	5	Describe the setting, locations, and relevant dates, including periods of recruitment, exposure, follow-up, and data collection.	p.4
	6	(a) Give the eligibility criteria, and the sources and methods of selection of participants. (b) For matched studies, give matching criteria.	pp.4–5
	7	Clearly define all outcomes, exposures, predictors, potential confounders, and effect modifiers. Give diagnostic criteria, if applicable.	pp.5–6
	8	For each variable of interest, give sources of data and details of methods of assessment (measurement). Describe comparability of assessment methods if more than one group.	p.5
	9	Describe any efforts to address potential sources of bias.	p.18 (Limitations)
	10	Explain how the study size was arrived at.	p.5 (all registry cases included)
	11	Explain how quantitative variables were handled in the analyses. If applicable, describe which groupings were chosen and why.	pp.5–6
	12	(a) Describe all statistical methods, including those used to control for confounding. (b) Describe any methods used to examine subgroups and interactions. (c) Explain how missing data were addressed. (d) If applicable, explain how loss to follow-up was addressed. (e) Describe any sensitivity analyses.	pp.5–6; updated to include regression diagnostics and robustness checks
Results	13	(a) Report numbers of individuals at each stage of study (e.g., numbers potentially eligible, examined for eligibility, confirmed eligible, included, completing follow-up, and analyzed). (b) Give reasons for non-participation at each stage. (c) Consider use of a flow diagram.	p.5; Table 1
	14	(a) Give characteristics of study participants (e.g.,	pp.6–8

Section/ Topic	Item No.	Recommendation	Reported on page(s)
		demographic, clinical, social) and information on exposures and potential confounders. (b) Indicate number of participants with missing data for each variable of interest. (c) Summarize follow-up time (e.g., average and total amount).	
	15	Report numbers of outcome events or summary measures over time.	pp.7–10 (Tables 2–4, Figures 1–9)
	16	(a) Give unadjusted estimates and, if applicable, confounder-adjusted estimates and their precision (e.g., 95% CI). Make clear which confounders were adjusted for and why they were included. (b) Report category boundaries when continuous variables were categorized. (c) If relevant, consider translating estimates of relative risk into absolute risk for a meaningful time period.	pp.10–12
	17	Report other analyses done—e.g., analyses of subgroups and interactions, and sensitivity analyses.	pp.12–13 (robustness checks)
Discussion	18	Summarize key results with reference to study objectives.	p.14
	19	Discuss limitations of the study, taking into account sources of potential bias or imprecision. Discuss both direction and magnitude of any potential bias.	pp.17–18
	20	Give a cautious overall interpretation of results considering objectives, limitations, multiplicity of analyses, results from similar studies, and other relevant evidence.	pp.14–18
	21	Discuss the generalisability (external validity) of the study results.	pp.18–19
Other information	22	Give the source of funding and the role of the funders for the present study.	p.20
